# Supplementary material for: KIT D816V Mast Cells Derived from Induced Pluripotent Stem Cells Recapitulate Systemic Mastocytosis Transcriptional Profile
Source: Int J Mol Sci. 2023 Mar 9;24(6):5275. doi: 10.3390/ijms24065275 (PMC10049485; doi:10.3390/ijms24065275)
Supplement: Supplementary file 1 [file ijms-24-05275-s001.zip › ijms-2223521-supplementary.pdf]

Supplemental data

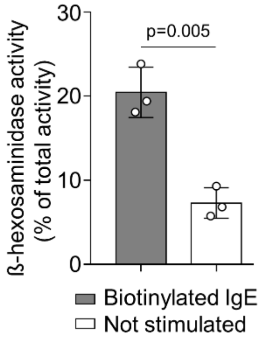

Supplemental Figure S1 – iPS cell-derived MC degranulate upon IgE crosslinking. Stimulated MC show higher β-hexosaminidase activity in the supernatant in comparison to unstimulated cells (Welch´s t-test, p= 0.005, n=3, 1 KIT D816V and 2 control).

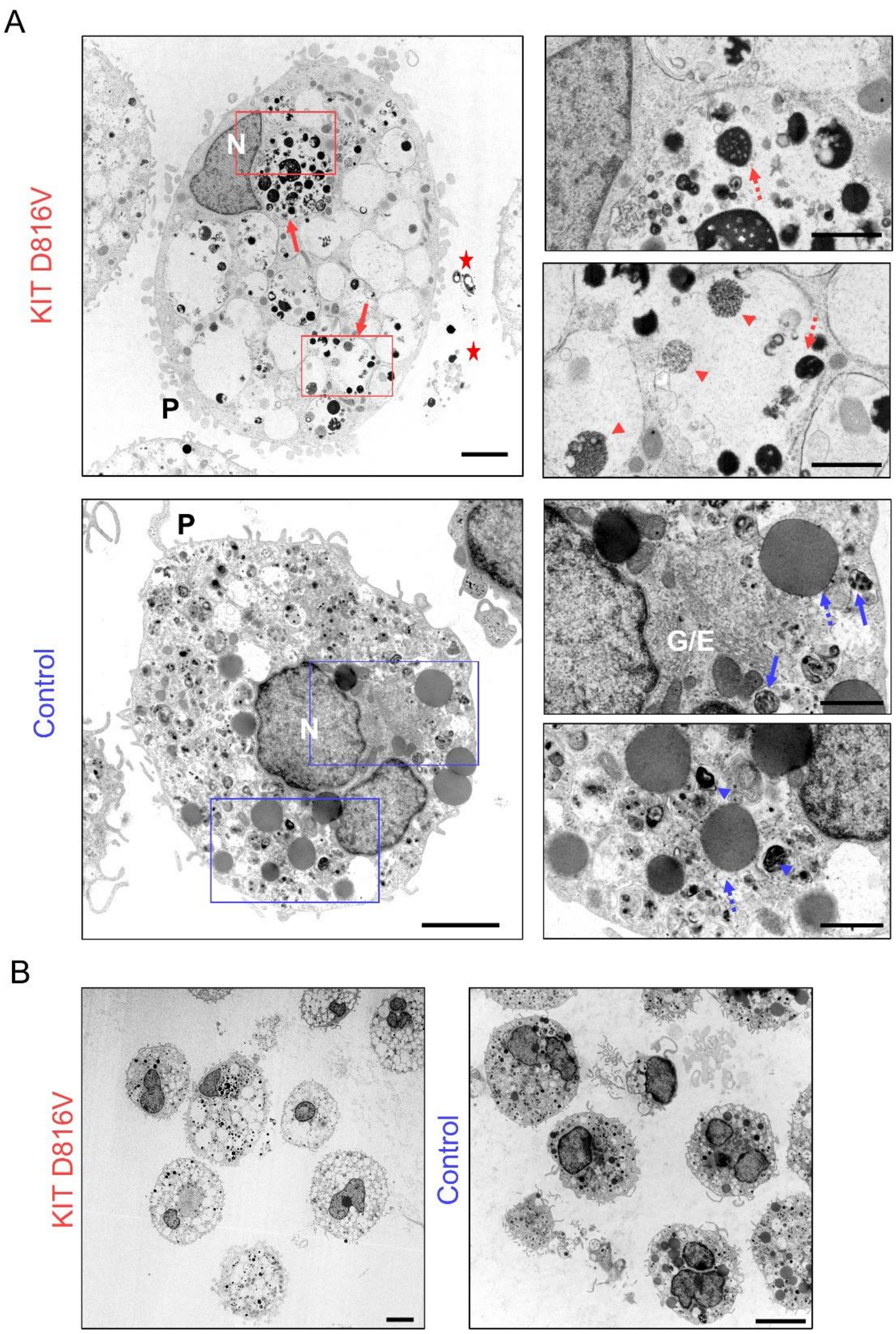

Supplemental Figure S2 – Ultrastructural characterization of iPS cell-derived MC. (A) Transmission electron microscopy (TEM) of KIT D816V MC (top panels, scale bar left panel: 2500 nm, right panels: 1000 nm) and control MC (bottom panels, scale bar left panel: 2500 nm, right panels: 1000 nm) as in Figure 3. KIT D816V MC display large, membrane-delimited compartments containing electron dense granules or granular remains with heterogenous morphology (top left panel, red arrows). These structures are commonly described in activated MC during the piecemeal degranulation process and may also be formed by fusion of several secretory granules. In line with an ongoing degranulation process, extracellular electron-dense granules are observed (red stars). Right panels show distinct electron dense granules (dashed red arrow) with variable granularity (red arrow heads). In control MC (bottom panels), characteristic membrane delimited secretory granules with dense cores in a translucent surrounding (blue arrows), larger electron dense/light granules (dashed blue arrows) and granules displaying electron-dense "scroll-like" arrangement (blue arrow heads) are observed. Nucleus (N), Golgi-associated endoplasmic reticulum (G/E) and thin cytoplasmic projections (P) are indicated. (B) MC displaying pronounced activated/degranulated phenotype (as described in A) are more commonly observed in the KIT D816V genotype (scale bar: 5000 nm).

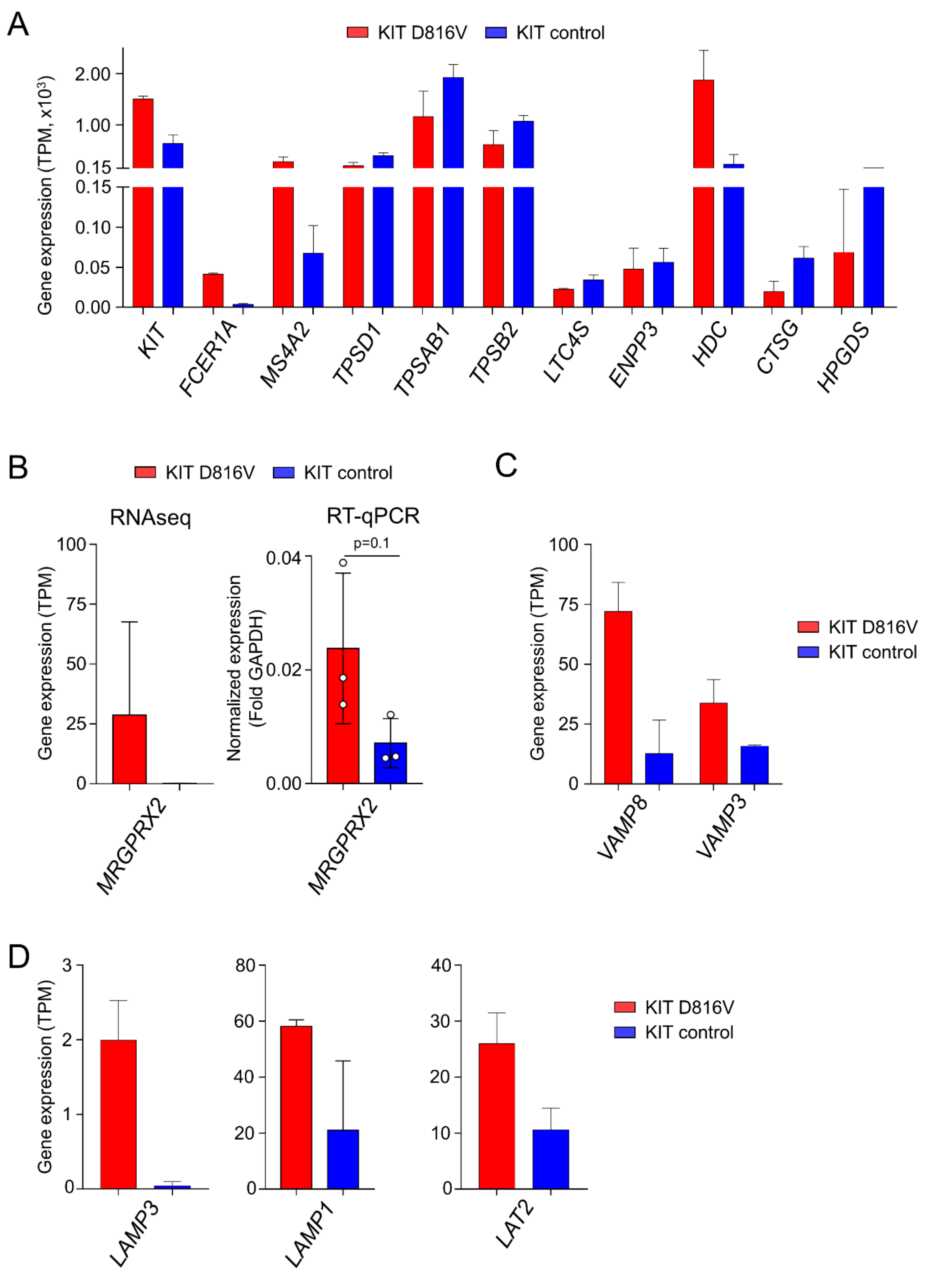

Supplemental Figure S3 – Global gene expression analysis of iPS cell-derived MC. (A) Expression values (transcripts per million, TPM) for 11 MC marker genes identified by global gene expression analysis of KIT D816V and control MC (please see Figure 4A). (B) KIT D816V MC show higher expression of *MRGPRX2*. Expression levels of *MRGPRX2* in global gene expression analysis (left panel, TPM) and by RT-qPCR (right panel, n=3 for KIT D816V and control, Welch´s t-test). (C) In global gene expression analysis, expression of granule membrane-associated proteins encoded by *VAMP8* and *VAMP3* is upregulated in KIT D816V MC. (D) Expression of degranulation-associated genes *LAMP3*, *LAMP1* and *LAT2* is also upregulated in KIT D816V MC as in panel C.

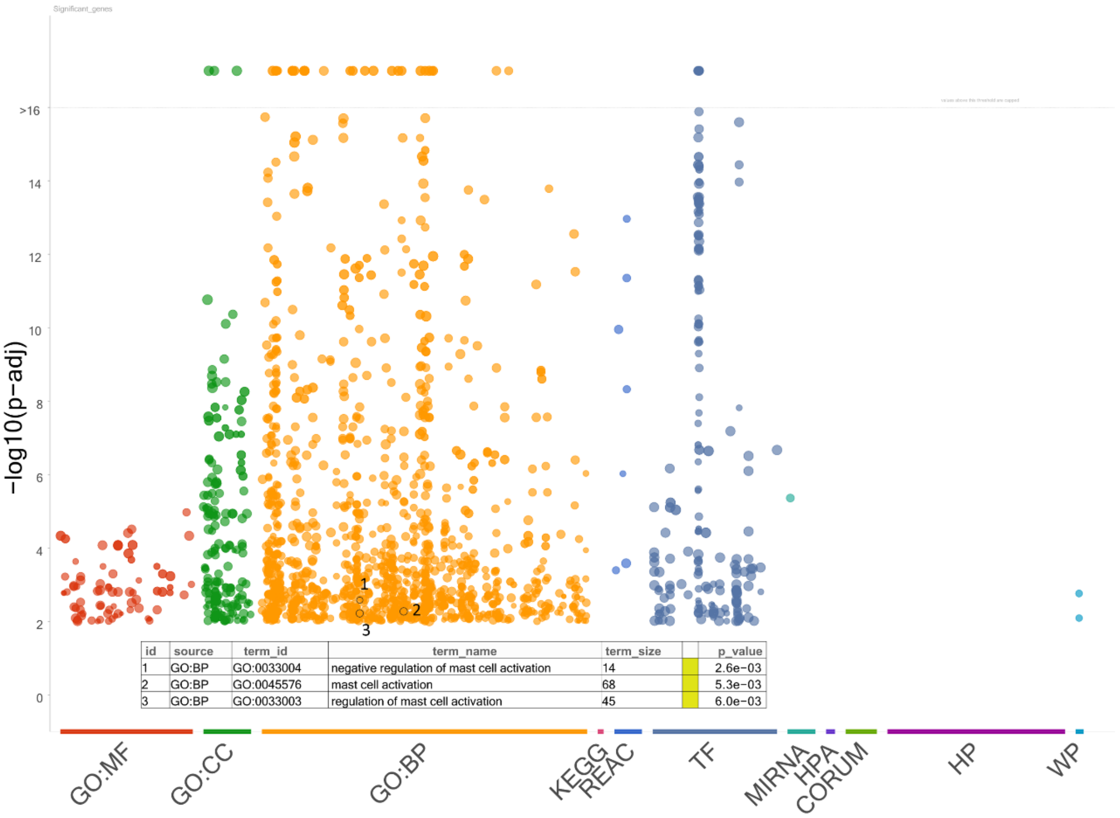

id	source	term_id	term_name	term_size		p_value
1	GO:BP	GO:0033004	negative regulation of mast cell activation	14		2.6e−03
2	GO:BP	GO:0045576	mast cell activation	68		5.3e−03
3	GO:BP	GO:0033003	regulation of mast cell activation	45		6.0e−03

Supplemental Figure S4 – GSEA of iPS cell-derived MC. KIT D816V MC show upregulated expression in pathways related to MC activation. Each dot represents a gene set inside a database (e.g., GO:MF, KEGG, WP) that is indicated by the different colors. The dots highlighted by 1, 2 and 3 show the significantly upregulated gene sets in KIT D816V MC that are involved in negative regulation of MC activation, MC activation, and regulation of MC activation, respectively.
